# Comparative transcriptomic analysis of a wing-dimorphic stonefly reveals candidate wing loss genes

**DOI:** 10.1186/s13227-019-0135-4

**Published:** 2019-09-06

**Authors:** Graham A. McCulloch, Andrew Oliphant, Peter K. Dearden, Andrew J. Veale, Charles W. Ellen, Jonathan M. Waters

**Affiliations:** 1 Department of Zoology, University of Otago, P.O. Box 56, Dunedin, 9054 New Zealand; 2 Genomics Aotearoa and Department of Biochemistry, University of Otago, P.O. Box 56, Dunedin, 9054 New Zealand; 3 Landcare Research, Private Bag 92170, Auckland Mail Centre, Auckland, 1142 New Zealand

**Keywords:** Vestigial winged, Wing development, Gene expression, *Zelandoperla fenestrata*

## Abstract

**Background:**

The genetic basis of wing development has been well characterised for model insect species, but remains poorly understood in phylogenetically divergent, non-model taxa. Wing-polymorphic insect species potentially provide ideal systems for unravelling the genetic basis of secondary wing reduction. Stoneflies (Plecoptera) represent an anciently derived insect assemblage for which the genetic basis of wing polymorphism remains unclear. We undertake quantitative RNA-seq of sympatric full-winged versus vestigial-winged nymphs of a widespread wing-dimorphic New Zealand stonefly, *Zelandoperla fenestrata*, to identify genes potentially involved in wing development and secondary wing loss.

**Results:**

Our analysis reveals substantial differential expression of wing-development genes between full-winged versus vestigial-winged stonefly ecotypes. Specifically, of 23 clusters showing significant similarity to *Drosophila* wing development-related genes and their pea aphid orthologues, nine were significantly upregulated in full-winged stonefly ecotypes, whereas only one cluster (*teashirt*) was substantially upregulated in the vestigial-winged ecotype.

**Conclusions:**

These findings suggest remarkable conservation of key wing-development pathways throughout 400 Ma of insect evolution. The finding that two Juvenile Hormone pathway clusters were significantly upregulated in vestigial-winged *Zelandoperla* supports the hypothesis that Juvenile Hormone may play a key role in modulating insect wing polymorphism, as has previously been suggested for other insect lineages.

## Background

The unparalleled diversity of insects is often attributed to the evolution of insect flight, ca. 400 Ma [1–5]. Specifically, wing evolution has carried numerous advantages, including an increased ability for insects to access novel resources and ecosystems, improved predator avoidance, and enhanced mate location [1]. This dispersal capacity has, however, been subsequently lost in numerous insect lineages, across almost all winged orders [6, 7].

The development and maintenance of wings requires significant energy expenditure, and some wing-dimorphic insect species thus exhibit apparent ‘trade-offs’ between flight-ability versus reproductive output [8–12]. Environmental conditions may also contribute to the evolution of wing-reduced lineages [6, 13], with flight loss particularly common in stable ecosystems that lack predators [14, 15]. Wing-reduced lineages are also disproportionally abundant in isolated areas, where there is a high level of mortality in dispersing individuals, and in areas where the energetic costs of flight are high [1, 16]. Likewise, wing loss is particularly common at higher altitudes [17], with recent studies implying that exposure, modulated by the alpine treeline, may be a key driver of insect wing reduction [18, 19].

Wings have been completely lost in many insect species, whereas some species comprise distinct winged and wingless ecotypes, often associated with differing environmental conditions [20–22]. As the distinct phenotypes of wing-dimorphic species have very similar genetic backgrounds, such taxa present ideal systems for exploring the molecular basis of wing loss. Previous studies of wing-dimorphic species have suggested that wing polymorphism can be genetically determined [23–27], controlled by environmentally driven gene expression (polyphenism; [11, 28–30]), include both genetic and environmental components [31], or be under the control of epigenetic factors [32, 33].

Given the range of environmental, genetic, and epigenetic factors that may contribute to wing polymorphism, elucidating the developmental basis of wing loss can be challenging. While the gene networks underpinning wing development have been well-characterised in model organisms such as *Drosophila melanogaster* [34, 35] and *Tribolium castaneum* [36], very little is known about the underlying molecular biology of wing development in non-model insect taxa. Several recent studies, however, have identified the key genes underlying wing development in non-model taxa by comparing gene-expression across winged and wingless morphs of wing polymorphic species (see Additional file 1: Table S1). While some portions of the key insect gene networks appear to be conserved among a variety of holometabolous and hemimetabolous taxa [37–40], the extent of their phylogenetic conservation across class Insecta remains unclear.

The New Zealand stonefly *Zelandoperla fenestrata* species group (hereafter simply referred to as *Z. fenestrata*) contains both full-winged and vestigial-winged ecotypes, with the wing-reduced phenotype particularly common at higher altitudes [20, 41]. The distinct morphotypes were traditionally considered distinct species (full-winged: *Zelandoperla fenestrata* or *Zelandoperla tillyardi*, vestigial-winged: *Zelandoperla pennulata*) [41], but more recent molecular studies demonstrate that vestigial-winged lineages have evolved independently on numerous occasions [20, 21]. In addition, a recent study identified several outlier loci strongly associated with vestigial versus full-winged ecotypes of *Z. fenestrata*, strongly suggesting a genetic basis for wing-length variation in this species [42].

In this study, we use quantitative RNA-seq to compare gene-expression patterns between the sympatric nymphs of full-winged versus vestigial-winged *Z. fenestrata*, to identify genes involved in wing development, wing growth and wing differentiation in this dimorphic species. Additionally, we assessed expression differences in candidate genes involved in the juvenile hormone (JH) pathway, as these genes have previously been demonstrated to play a role in insect wing polymorphisms [11, 28]. We identified genes that were significantly differentially expressed between vestigial-winged and full-winged ecotypes, with a particular focus on genes known to be involved in the wing-development networks.

## Results

### Transcriptome sequencing, assembly, and annotation

Sequencing yielded 204,011,700 125-bp paired reads. After initial adapter trimming and quality filter, 147,396,767 reads remained (Additional file 1: Table S2). These raw RNA-seq reads have been deposited in the NCBI repository under BioProject PRJNA525904. De novo assembly using Trinity generated 552,851 transcripts hierarchically clustered into 442,924 Trinity ‘genes’, with a mean length of 587.6 bp and an N50 of 887 bp (Additional file 1: Table S2). 98.6% of BUSCO proteins were identified as full-length proteins, with only 1.0% fragmented and 0.4% missing (Additional file 1: Table S2). Over 84% of input reads aligned as proper pairs one or more times, and the overall alignment was over 97% (Additional file 1: Table S2). TransDecoder predicted open reading frames in 108,209 transcripts and BLASTp annotated over 70% of these ORFs (UniProt/Swiss-Prot database) (Additional file 1: Table S3).

Corset clustering reduced the number of transcripts to 227,791, which were assigned to 140,592 clusters with a mean transcript length of 1023.5 bp and an N50 of 1668 bp (Additional file 1: Table S2). 98.5% of BUSCOs were found to be complete with 0.7% missing and 0.8% fragmented (Additional file 1: Table S2). 84% of input reads aligned concordantly one or more times, and the overall alignment was over 96% (Additional file 1: Table S2).

### Genes differentially expressed across the full-winged and vestigial-winged ecotypes

DE analysis identified 1042 clusters differentially expressed at FDR ≤ 0.01 and LogFC ≥ 1.5 or ≤ − 1.5. Of the 1042 DE clusters, 511 contained TransDecoder-predicted longest ORFs which were annotated by emapper. Of these, 332 clusters were more abundant in full-winged individuals, whilst 179 clusters were more highly expressed in vestigial-winged notum (Fig. 2). Forty-three clusters were annotated with GO:0035220, biological process ‘wing disc development’; 17 of which were more highly expressed in full-winged notum, whilst 26 were more abundant in vestigial-winged notum. All clusters with FDR ≤ 0.01 and LogFC ≥ 1.5 or ≤ − 1.5, including annotations, are provided in Additional file 2: Table S4.

### Identification and expression of wing development genes

Twenty-three clusters with significant similarity to *Drosophila* wing development-related genes and their pea aphid orthologues were identified, including genes in each of the four wing development pathways, as well as homeobox genes (Table 1). As a result of low numbers of reads aligning to the transcripts of one of these clusters, statistics and expression data were available for only 22 clusters. Just over half of these clusters were not significantly differentially expressed across ecotypes (Fig. 3a). However, nine clusters were significantly more expressed in the notum of full-winged individuals (at FDR ≤ 0.01, LogFC ≥ 1.5), including *wingless*, *nubbin*, *distalless*, *hedgehog*, *ventral veins lacking*, and *engrailed* (Fig. 3a). By contrast only a single cluster (*teashirt*) was upregulated in the vestigial-winged ecotype (Fig. 3a). DE analysis also revealed two clusters with known roles in wing development; *optomotor*-*blind protein* and a *frizzled* receptor family member. Both were more highly expressed in full-winged individuals (Fig. 3b).

**Table 1:** Wing development genes predicted in the notum of vestigial- and full-winged *Zelandoperla fenestrata*

Wing development gene	Top (NCBI) blast hit			
	e-Value	Species	Gene	Accession
Engrailed (en)	2.00E−17	*Drosophila miranda*	Engrailed	XM_017294233.1
Hedgehog (hh)	4.00E−180	*Neodiprion lecontei*	Hedgehog	XM_015659074.1
Cubitus interruptus (ci)	0.00E+00	*Zootermopsis nevadensis*	Cubitus interruptus	XM_022064111.1
Patched (ptc)	0.00E+00	*Cryptotermes secundus*	Patched	XM_023869672.1
Decapentaplegic (dpp)	1.00E−130	*Agrilus planipennis*	Decapentaplegic	XM_018469193.1
Spalt major (sal)	0.00E+00	*Tribolium castaneum*	Spalt-major	XM_008195490.2
Apterous (ap)	2.00E−75	*Cryptotermes secundus*	Apterous	XM_023854991.1
Apterous-like	1.00E−40	*Zootermopsis nevadensis*	Apterous-like	XM_022074071.1
Notch	0.00E+00	*Cryptotermes secundus*	Notch	XM_023868524.1
Serrate (Ser)	0.00E+00	*Cryptotermes secundus*	Jagged	XM_023862401.1
Wingless (wg1)	0.00E+00	*Periplaneta americana*	Wingless	KJ680328.1
Wingless (wg16)	0.00E+00	*Cryptotermes secundus*	Wnt-16	XM_023857064.1
Wingless (wg7)	5.00E−165	*Cryptotermes secundus*	Wnt-7	XM_023860077.1
Wingless (wg6)	0.00E+00	*Cryptotermes secundus*	Wnt-6	XM_023845703.1
Wingless (wg5)	0.00E+00	*Zootermopsis nevadensis*	Wnt-5	XM_022080506.1
Distalless (Dll)	1.00E−94	*Cryptotermes secundus*	Distal-less	XM_023852254.1
Vestigial (vg)	7.00E−50	*Cryptotermes secundus*	Vestigial	XM_023854945.1
Anntenapedia (Antp)	6.00E−52	*Cryptotermes secundus*	Antennapedia-like	XR_002842332.1
Ultrabithorax (Ubx)	4.00E−111	*Nilaparvata lugens*	Ultrabithorax	KR869786.1
Homothorax (hth)	0.00E+00	*Zootermopsis nevadensis*	Homothorax	XM_022058904.1
Teashirt (tsh)	2.00E−173	*Zootermopsis nevadensis*	Tiptop	XM_022075564.1
Nubbin (nub)	1.00E−67	*Blattella germanica*	Nubbin	LT216433.1
Ventral veins lacking (vvl)	8.00E−172	*Zootermopsis nevadensis*	POU domain protein CF1A	XM_022061685.1

In addition to wing development genes (above), a juvenile hormone acid methyltransferase cluster was identified as differentially expressed between the two ecotypes, being significantly upregulated in the vestigial-winged samples (Fig. 3b). A juvenile hormone esterase cluster was also significantly differentially expressed between vestigial-winged and full-winged ecotypes, being more abundant in vestigial-winged ecotypes (Fig. 3b).

## Discussion

**Fig. 1:**
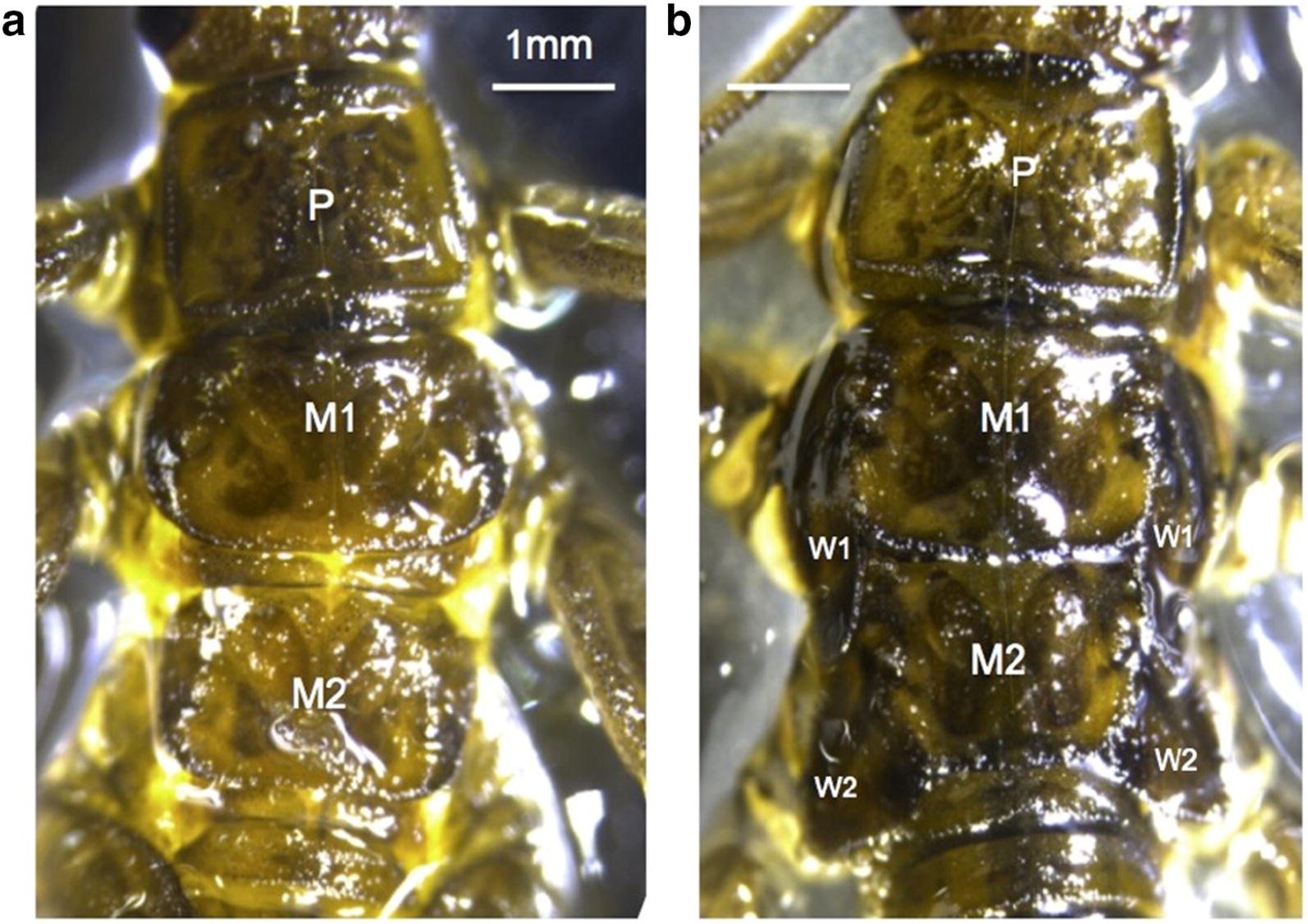
Dorsal view of *Z. fenestrata* nymphs sequenced in this study illustrating the extent of wing-bud development in **a** the vestigial-winged ecotype, and **b** the full-winged ecotype. *P* pronotum, *M1* mesonotum, *M2* metanotum, *W1/W2* wing-buds

**Fig. 2:**
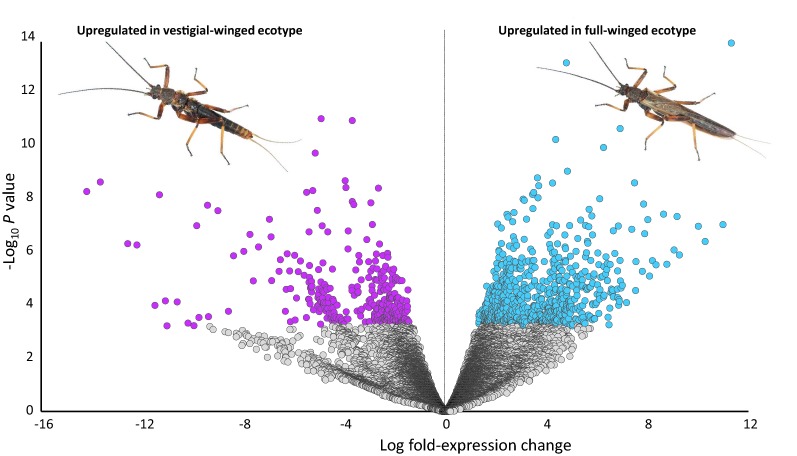
Volcano plot displaying differentially expressed genes in the notum of full-winged and vestigial-winged *Zelandoperla fenestrata*. The x-axis displays the log fold-expression change, with negative values indicating increased expression in the vestigial-winged ecotype, and positive values indicating increased expression in the full-winged ecotype. The *y*-axis displays the −log_10_ adjusted *p* value. Genes that were significantly more expressed (FDR ≤ 0.01) are coloured (full-winged ecotype = cyan, vestigial-winged ecotype = magenta)

**Fig. 3:**
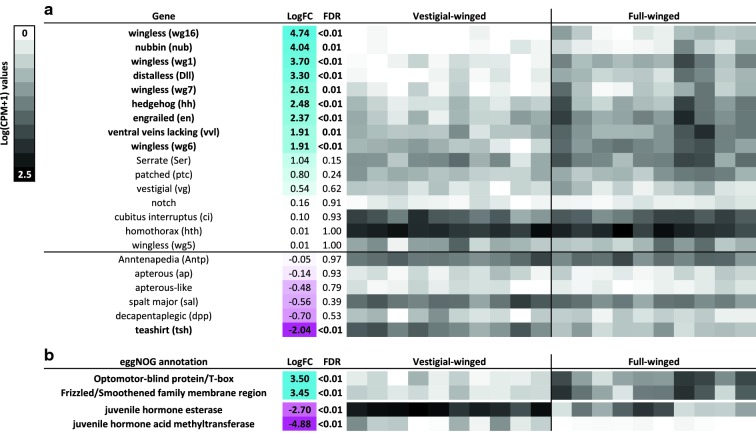
Comparative expression of **a** wing development genes and **b** additional genes of interest in the notum of full-winged versus vestigial-winged ecotypes. Genes in bold are significantly differentially expressed across ecotypes. Positive LogFC values indicate the gene is more expressed in full-winged ecotypes, with the intensity of the cyan shading indicating the extent of increased expression. Negative LogFC values indicate the gene is more expressed in vestigial-winged ecotypes, with the intensity of magenta shading indicating the extent of increased expression. Shading in columns to the right indicate the expression of each gene in individual samples (see key)

In this study, we compared the expression of genes in the notum of full-winged versus vestigial-winged ecotypes of the wing-dimorphic stonefly *Zelandoperla fenestrata.* The former specimens had prominent wing-bud development, whereas the latter did not (Fig. 1). We identified over 20 clusters with significant similarity to genes previously implicated in wing development in *Drosophila melanogaster*. Over 1000 clusters were significantly differentially expressed among the two ecotypes, including numerous clusters annotated as genes involved in key wing development pathways (Figs. 2, 3). We discuss the implications of these findings, particularly in reference to identifying the key gene/s potentially involved in wing loss, below.

Some of the clusters with differential expression between full-winged and vestigial-winged forms of *Z. fenestrata* stoneflies are also critical components of wing patterning in *D. melanogaster*. While it is unclear how stonefly wing development differs from *Drosophila*, the key genes appear conserved, and the common ancestry of these insect lineages implies that some aspects of the patterning system are likely conserved, especially those involved in initial patterning. This finding suggests a remarkable conservation of these key developmental pathways, as the ancestors of Plecoptera and Diptera diverged ca. 400 Ma [4].

About half of these candidate wing-development genes were not significantly differentially expressed across the two ecotypes (Fig. 3a). This result is unsurprising, as many of these genes are highly pleiotropic, having additional important developmental roles [40]. Several key wing-development genes were, however, significantly differentially expressed across vestigial-winged and full-winged ecotypes (Fig. 3a). The prominent wing-buds in the full-winged specimens indicate that wing development is already well underway; it, therefore, seems likely that the lower expression level of these wing-development genes in the vestigial-winged ecotype may represent the down-stream consequences of wing reduction, rather than representing the underlying causes of wing reduction.

In *Drosophila*, three sets of genes act to define the anterior–posterior, the dorsoventral and the proximo-distal axes (reviewed by [43, 44]). The anterior–posterior axis is defined by the expression of *engrailed* [45]. *Engrailed* causes the expression of *hedgehog* [46], which diffuses across the anterior–posterior boundary of the wing, to activate *decapentaplegic* [47, 48]. Expression of *engrailed* and *hedgehog* is reduced in vestigial-winged compared to full-winged stoneflies, as is the expression of *optomotor*-*blind* (a gene acting downstream of *decapentaplegic*), confirming these genes play crucial roles in stonefly wing development.

The dorsoventral axis of the *Drosophila* wing is patterned by *wnt* signalling molecules, especially *wingless* (wg1) [49–52]. *Wingless* and three other *wg* molecules (wg 6, 7, and 16) have reduced expression in vestigial-winged stoneflies, as do their receptors, *frizzled*. A downstream patterning factor responding to *wingless*, *ventral*-*veinless* (also known as *drifter*) [53] is also significantly lower in expression in vestigial-winged stoneflies.

Proximo-distal growth of the *Drosophila* wing is regulated by two key genes, *teashirt* and *nubbin* [54]. *Nubbin* is down-regulated in vestigial-winged stoneflies, while *teashirt* is up-regulated. *Teashirt* is known to be highly expressed in the notum and wing-hinge of *Drosophila* [55], so increased expression was not unexpected in the vestigial-winged nymphs given the slightly higher proportion of notum tissue in the vestigial-winged extractions (see Fig. 1). However, this slightly higher proportion of notum tissue is unlikely to explain the fourfold change in expression we observed (Fig. 3a). While it is not clear what the consequences for wing outgrowth of these changes in gene expression are, they imply mechanisms of proximo-distal axis growth of the wing differ between full-winged and vestigial-winged forms. In *Drosophila*, repression of *teashirt* is required for the initiation of wing development [55]. That this gene is upregulated in vestigial-winged stoneflies implies that down-regulation may not occur, potentially providing a proximate mechanism (rather than the ultimate cause) of wing reduction.

Differences in gene expression between full-winged versus vestigial-winged forms of stonefly encompass mechanisms that define and pattern all three wing axes, implying that all aspects of wing growth and patterning may be reduced in vestigial-winged stoneflies. Given the relatively rapid evolution of this trait (see [19]), it seems very unlikely that simultaneous changes across all of these networks underlie the causative changes in the evolution of vestigial-winged forms. We, therefore, suggest that the causative changes may lie upstream of wing patterning and growth, perhaps in juvenile hormone (JH) signalling. JHs regulate several key processes in insect physiology [56], and have previously been associated with wing polymorphism in both Orthoptera [28, 57] and Hemiptera [58, 59].

Two JH pathway clusters were significantly upregulated in the vestigial-winged ecotype, suggesting JH may play a role in modulating wing polymorphism in *Z. fenestrata*. JH levels in insects can vary both within and across developmental instars, with JH levels typically lower in later instars, but with levels also dropping significantly directly before moulting [56]. Populations of *Z. fenestrata* may comprise individuals at a variety of developmental stages, as the species has an extended emergence ‘window’ (see [60]). Although the wild-caught nymphs used in this study were all late instars, we were unable to *tightly* control for their developmental stage, and as a result, some variation in the expression of key JH-related genes might be expected among individuals (e.g. within ecotypes). For instance, the finding that JH acid methyltransferase expression varied substantially among vestigial-winged specimens (Fig. 3b) might potentially reflect minor developmental differences among these specimens. However, there is no evidence for systematic developmental asynchrony among these ecotypes where they co-occur, and indeed there are several localities at which we have simultaneously collected both full-winged and vestigial-winged adults [19, 42]. Systematic differences among ecotypes in the expression of JH-related genes are thus unlikely to result solely from (minor) differences in developmental stage. For instance, JH acid methyltransferase expression is *consistently* low across all full-winged specimens (despite likely developmental variation within this ecotype, see Fig. 3b), but variable among vestigial-winged individuals. Similarly, JH esterase expression is *consistently* high across all vestigial-winged specimens, but is highly variable in full-winged individuals. In both cases, the extensive differentiation among ecotypes is unlikely to reflect developmental timing, suggesting that the repression of these genes may play an important role in *Z. fenestrata* wing development.

In addition to the extensive evidence for effects of JHs on insect wing development, these genes have previously been associated with delayed metamorphosis [61], and variation in several other insect life-history traits (e.g. fecundity; [62]). It is thus conceivable that JH could simultaneously modulate both wing phenotype variation and additional developmental differentiation between high-altitude and low-altitude stonefly lineages (such as differences in body size and emergence timing, see [60, 63]). Future molecular and ecological studies will aim to unravel the potential role of JH in explaining ecotypic variation within and among wing-polymorphic stonefly taxa.

## Conclusion

Our study reveals differential expression of wing-development genes between closely related full-winged and vestigial-winged stonefly ecotypes, consistent with previous data from *Drosophila*, suggesting remarkable conservation of key wing development pathways throughout 400 Ma of insect evolution. The significant gene expression differentiation observed among stonefly ecotypes potentially provides fertile new directions for future evolutionary research on insect wing reduction.

## Methods

### Sample collection

Late instar *Z. fenestrata* nymphs were collected by hand from beneath rocks in the stream at an altitude of 670 m in Lug Creek, Rock and Pillar Range, Otago, New Zealand, in July 2017. This site is just above the alpine tree line, and is part of a narrow zone where full-winged and vestigial-winged ecotypes are found in sympatry [19]. Nymphs were stored in containers in water from their natal stream, and kept cool in an ice bath while they were returned to the laboratory. Nymphs were characterised as either full-winged or vestigial-winged based on clear differences in wing-bud development (Fig. 1), as previous work has indicated that these two wing-bud development types are correlated with the distinct adult ecotypes [19, 41, 42].

### RNA extraction

Ten nymphs from each of the vestigial-winged and full-winged ecotypes were extracted and sequenced. As sufficient quantities of RNA could not readily be obtained from the wing-buds (alone) of vestigial winged nymphs (as their wing-buds are extremely small, see Fig. 1), we instead extracted RNA from the complete notum (including wing-buds) of both ecotypes. This consistent approach allowed us to directly compare gene expression across full-winged and vestigial-winged ecotypes, although we note the ratio of wing-bud tissue to notum tissue differed across vestigial-winged and full-winged specimens (see Fig. 1). Nymphs were immobilised briefly on dry ice, then pinned through the head and abdomen under a binocular dissection scope before the mesonotum (M1; Fig. 1) and metanotum (M2; Fig. 1) were removed as a single sample. A shallow incision using a razorblade was made across the dorsal surface between the protonotum and M1. A lateral incision was then made on each side separating M1 and M2 from the meso- and metapleuron. A final incision between M2 and the first tergal segment was used to free M1 and M2. Care was taken to remove any fat bodies or viscera that were attached centrally to M1 or M2 to maintain consistency between samples. Once dissected, the notum samples were immediately frozen on dry ice and stored at − 80 °C.

Tissue was homogenised with hard plastic homogenising probes in RTL buffer (supplied as part of the Qiagen RNeasy kit) using an Omni Tissue Homogenizer. RNA isolation was then completed following the protocol supplied with the RNeasy kit (Qiagen), including an on-column DNAse digestion. Once extracted and washed, samples were eluted with 35 μL of RNAse-free water and then stored at − 80 °C.

### Library preparation

The 260/230 and 260/280 ratios were examined using a NanoDrop spectrophotometer (Thermo Scientific), and the concentrations were assessed using a Qubit fluorometer (Thermo-Fisher Scientific) with the RNA HS kit. RNA integrity was also assessed on a 1.5% agarose gel after electrophoresis. We used a poly-A capture to select for mRNA, and created a library using a TruSeq Stranded mRNA Library Prep Kit (Illumina), with each individual tagged to separate reads bioinformatically after sequencing. Library quality was assessed using the Agilent 2100 Bioanalyzer and TBS 380 Fluorometer (Turner Biosystems, Sunny-vale, CA, USA), and all libraries were paired-end sequenced (2 × 125 bp) on a single lane of an Illumina HiSeq 2500.

### Transcriptome assembly

The combined vestigial-winged- and full-winged-notum transcriptome was de novo assembled using Trinity v2.8.4 [64, 65]. Reads were quality trimmed using Trimmomatic, run as part of the Trinity package with default settings [66, 67], and normalised in silico prior to assembly. Descriptive statistics were calculated using TrinityStats.pl. Transcriptome completeness was assessed using BUSCO v3.0.2 (an arthropod_odb9 database of 1066 BUSCOs covering 60 species; [68, 69]) and RNA-seq read representation following the trinityrnaseq-wiki (github.com/trinityrnaseq/trinityrnaseq/wiki). Transcript clusters, clustered using Corset (see below), were quality checked in the same way.

The Trinity transcriptome was annotated using Trinotate v3.1.1 [70] and associated packages (TransDecoder v5.5.0, SQLite, blast + v, hmmer v3.2.1, Rnammer v1.2, signalP v4.1, tmhmm v2.0, trinotateR). Annotation of differentially expressed clusters (see below) used emapper-1.0.3 [71] based on eggNOG orthology data [72] with sequence searches performed using HMM and diamond [73, 74]. ‘Contigs of interest’ (i.e. differentially expressed clusters of contigs) were extracted from the Trinity transcriptome fasta file using ‘fetchClusterSeqs.py’ script (github.com/Adamtaranto/Corset-tools) and protein sequences predicted using TransDecoder v 5.5.0. Annotation of the longest ORFs was performed using emapper and two methods: HMMER, using the arthropod protein database artNOG, and diamond, using the eggNOG protein database.

### Identification of differentially expressed transcripts

Differential gene expression between vestigial-winged and full-winged individuals was assessed using Corset v1.07 [75] and with read alignment using bowtie2 (v2.3.4.1) with multi-mapping enabled. Statistical analysis used edgeR in R i386 3.5.1 [76]. Analysis followed the Corset wiki (github.com/Oshlack/Corset/wiki).

### Genes of interest

In addition to identifying differentially expressed genes, expression data for genes with known roles in wing development were sought. Local blast searches were done using *Drosophila* wing development-related genes and orthologues from the pea aphid, *Acyrthosiphon pisum* [40], as query terms (taken from the NCBI website: http://www.ncbi.nlm.nih.gov), and using BioEdit software [77]. Searches were done against clusters (Corset clustered transcripts).

## Supplementary information

**Additional file 1: Table S1.** Recent studies examining the genetic basis for wing development in Pterygota. **Table S2**. Descriptive statistics for *Zelandoperla fenestrata* notum transcriptome. **Table S3.** Annotation statistics for *Zelandoperla fenestrata* notum transcriptome.

**Additional file 2: Table S4.** All gene-clusters significantly differentially expressed across full-winged and vestigial-winged ecotypes (FDR ≤ 0.01), including annotations.

## Data Availability

The dataset supporting the conclusions of this article is available in the NCBI repository under BioProject PRJNA525904.
