# Supplementary material for: Comparative transcriptomic analysis of a wing-dimorphic stonefly reveals candidate wing loss genes
Source: EvoDevo. 2019 Sep 6;10:21. doi: 10.1186/s13227-019-0135-4 (PMC6728979; doi:10.1186/s13227-019-0135-4)
Supplement: Supplementary file 1 — Additional file 1: Table S1. Recent studies examining the genetic basis for wing development in Pterygota. Table S2. Descriptive statistics for Zelandoperla fenestrata notum transcriptome. Table S3. Annotation statistics for Zelandoperla fenestrata notum transcriptome. [file 13227_2019_135_MOESM1_ESM.docx]

| **Order: Family** | **Species** | **Study Type** | **Reference** |
| --- | --- | --- | --- |
| Blattodea: Ectobiidae | *Blattella germanica.* | segment comparison | (1) |
| Coleoptera: Carabidae | *Pogonus chalceus* | polymorphism | (2) |
| Coleoptera: Buprestidae | *Ostrinia furnacalis* | species comparison | (3) |
| Coleoptera: Coccinellidae | *Adalia bipunctata* | polymorphism | (4) |
| Diptera: Drosophilidae | *Drosophila melanogaster* | polymorphism | (5-9) |
| Diptera: Tephritidae | *Bactrocera correcta* | polymorphism | (10) |
| Diptera: Tephritidae | *Bactrocera dorsalis* | polymorphism | (10) |
| Hemiptera: Aphidoidea | *Acyrthosiphon pisum* | polymorphism | (8, 11, 12) |
| Hemiptera: Aphidoidea | *Toxoptera citricida* | polymorphism | (13) |
| Hemiptera: Coccidae | *Ericerus pela* | polyphenism | (14) |
| Hemiptera: Delphacidae | *Nilaparvata lugens* | polymorphism | (3, 15, 16) |
| Hemiptera: Delphacidae | *Sogatella furcifera* | polymorphism | (17, 18) |
| Hemiptera: Gerridae | *Gigantometra gigas* | polymorphism | (19) |
| Hemiptera: Lygaeidae | *Oncopeltus fasciatus* | RNAi | (20) |
| Hymenoptera: Chalcidoidea | *Ceratosolen solmsi* | polyphenism | (21) |
| Hymenoptera: Formicidae | *Crematogaster lineolata,* | polyphenism | (22) |
| Hymenoptera: Formicidae | *Lasius niger* | polyphenism | (22) |
| Hymenoptera: Formicidae | *Mystrium oberthueri* | polyphenism | (23) |
| Hymenoptera: Formicidae | *Mystrium rogeri* | polyphenism | (23) |
| Hymenoptera: Formicidae | *Pheidole morrisi* | polyphenism | (24, 25) |
| Hymenoptera: Formicidae | *Polistes metricus* | polyphenism | (26) |
| Hymenoptera: Formicidae | *Tetramorium caespitum,* | polyphenism | (22) |
| Lepidoptera: Bombycidae | *Bombyx mori* | polymorphism | (27) |
| Odonata: Zygoptera | *Megaloprepus caerulatus* | polymorphism | (28) |
| Orthoptera: Acrididae | *Locusta migratoria* | species comparison/ developmental | (3, 29) |
| Orthoptera: Gryllidae | *Gryllus firmus* | polymorphism | (30) |

**Table S1.** Recent studies examining the genetic basis for wing development in Pterygota

1. Elias-Neto M, Belles X. Tergal and pleural structures contribute to the formation of ectopic prothoracic wings in cockroaches. Royal Soc Open Sci. 2016;3:160347.

2. Van Belleghem SM, Roelofs D, Van Houdt J, Hendrickx F. De novo transcriptome assembly and SNP discovery in the wing polymorphic salt marsh beetle *Pogonus chalceus* (Coleoptera, Carabidae). PLoS ONE. 2012;7:e42605.

3. Liu S, Wei W, Chu Y, Zhang L, Shen J, An C. De novo transcriptome analysis of wing development-related signaling pathways in *Locusta migratoria manilensis* and *Ostrinia furnacalis* (Guenee). PLoS ONE. 2014;9:e106770.

4. Lommen ST, Saenko SV, Tomoyasu Y, Brakefield PM. Development of a wingless morph in the ladybird beetle, *Adalia bipunctata*. Evol Dev. 2009;11:278-89.

5. Dworkin I, Anderson JA, Idaghdour Y, Parker EK, Stone EA, Gibson G. The effects of weak genetic perturbations on the transcriptome of the wing imaginal disc and its association with wing shape in *Drosophila melanogaster*. Genetics. 2011;187:1171-84.

6. O’Keefe D, Thomas SR, Bolin K, Griggs E, Edgar BA, Buttitta LA. Combinatorial control of temporal gene expression in the *Drosophila* wing by enhancers and core promoters. BMC Genomics. 2012;13:498.

7. Organista MF, Martin M, de Celis JM, Barrio R, López-Varea A, Esteban N, et al. The Spalt transcription factors generate the transcriptional landscape of the *Drosophila melanogaster* wing pouch central region. PLoS Genet. 2015;11:e1005370.

8. Agnel S, da Rocha M, Robichon A. Transcriptome Profiling of Neurosensory Perception Genes in Wing Tissue of Two Evolutionary Distant Insect Orders: Diptera (*Drosophila melanogaster*) and Hemiptera (*Acyrthosiphon pisum*). J Mol Evol. 2017;85:234-45.

9. Dye NA, Popović M, Spannl S, Etournay R, Kainmüller D, Ghosh S, et al. Cell dynamics underlying oriented growth of the *Drosophila* wing imaginal disc. Development. 2017;144:4406-21.

10. Guo S, Zhao Z, Liu L, Li Z, Shen J. Comparative transcriptome analyses uncover key candidate genes mediating flight capacity in *Bactrocera dorsalis* (Hendel) and *Bactrocera correcta* (Bezzi)(Diptera: Tephritidae). Int J Mol Sci. 2018;19:396.

11. Vellichirammal NN, Madayiputhiya N, Brisson JA. The genomewide transcriptional response underlying the pea aphid wing polyphenism. Mol Ecol. 2016;25:4146-60.

12. Brisson JA, Ishikawa A, Miura T. Wing development genes of the pea aphid and differential gene expression between winged and unwinged morphs. Insect Mol Biol. 2010;19:63-73.

13. Shang F, Ding B-Y, Xiong Y, Dou W, Wei D, Jiang H-B, et al. Differential expression of genes in the alate and apterous morphs of the brown citrus aphid, *Toxoptera citricida*. Sci Rep. 2016;6:32099.

14. Yang P, Chen X-M, Liu W-W, Feng Y, Sun T. Transcriptome analysis of sexually dimorphic Chinese white wax scale insects reveals key differences in developmental programs and transcription factor expression. Sci Rep. 2015;5:8141.

15. Li K-y, Hu D-b, Liu F-z, Man L, Liu S-y, Jing Z, et al. Wing patterning genes of *Nilaparvata lugens* identification by transcriptome analysis, and their differential expression profile in wing pads between brachypterous and macropterous morphs. J Integr Agric. 2015;14:1796-807.

16. Xue J, Bao Y-Y, Li B-l, Cheng Y-B, Peng Z-Y, Liu H, et al. Transcriptome analysis of the brown planthopper *Nilaparvata lugens*. PLoS ONE. 2010;5:e14233.

17. Liang A-W, Zhang H, Lin J, Wang F-H. De novo assembly and analysis of the white-backed planthopper (*Sogatella furcifera*) transcriptome. J Insect Sci. 2018;18.

18. Liang Z-Q, Song S-Y, Liang S-K, Wang F-H. Analysis of differential proteins in two wing-type females of *Sogatella furcifera* (Hemiptera: Delphacidae). J Insect Sci. 2016;16:35.

19. Sun X-y, Wang Y-h, Dong Z-e, Wu H-y, Chen P-p, Xie Q. Identifying differential gene expression in wing polymorphism of adult males of the largest water strider: de novo transcriptome assembly for *Gigantometra gigas* (Hemiptera: Gerridae). J Insect Sci. 2018;18:17.

20. Medved V, Marden JH, Fescemyer HW, Der JP, Liu J, Mahfooz N, et al. Origin and diversification of wings: Insights from a neopteran insect. Proc Natl Acad Sci USA. 2015;112:15946-51.

21. Sun B-F, Li Y-X, Jia L-Y, Niu L-H, Murphy RW, Zhang P, et al. Regulation of transcription factors on sexual dimorphism of fig wasps. Sci Rep. 2015;5:10696.

22. Shbailat SJ, Abouheif E. The wing‐patterning network in the wingless castes of Myrmicine and Formicine ant species is a mix of evolutionarily labile and non‐labile genes. J Exp Zool B Mol Dev Evol. 2013;320:74-83.

23. Béhague J, Fisher BL, Péronnet R, Rajakumar R, Abouheif E, Molet M. Lack of interruption of the gene network underlying wing polyphenism in an early‐branching ant genus. J Exp Zool B Mol Dev Evol. 2018;330:109-17.

24. Shbailat SJ, Khila A, Abouheif E. Correlations between spatiotemporal changes in gene expression and apoptosis underlie wing polyphenism in the ant *Pheidole morrisi*. Evol Dev. 2010;12:580-91.

25. Abouheif E, Wray GA. Evolution of the gene network underlying wing polyphenism in ants. Science. 2002;297:249-52.

26. Berens AJ, Hunt JH, Toth AL. Comparative transcriptomics of convergent evolution: different genes but conserved pathways underlie caste phenotypes across lineages of eusocial insects. Mol Biol Evol. 2014;32:690-703.

27. Zhang J, Blessing D, Wu C, Liu N, Li J, Qin S, et al. Comparative transcriptomes analysis of the wing disc between two silkworm strains with different size of wings. PLoS ONE. 2017;12:e0179560.

28. Feindt W, Oppenheim SJ, DeSalle R, Goldstein PZ, Hadrys H. Transcriptome profiling with focus on potential key genes for wing development and evolution in *Megaloprepus caerulatus*, the damselfly species with the world's largest wings. PLoS ONE. 2018;13:e0189898.

29. Zhao X, Gou X, Qin Z, Li D, Wang Y, Ma E, et al. Identification and expression of cuticular protein genes based on Locusta migratoria transcriptome. Sci Rep. 2017;7:45462.

30. Vellichirammal NN, Zera AJ, Schilder RJ, Wehrkamp C, Riethoven J-JM, Brisson JA. De novo transcriptome assembly from fat body and flight muscles transcripts to identify morph-specific gene expression profiles in *Gryllus firmus*. PLoS ONE. 2014;9:e82129.

**Table S2**. Descriptive statistics for *Zelandoperla fenestrata* notum Trinity transcriptome and Corset clusters

| **Statistic** | **Trinity** | **Corset** |
| --- | --- | --- |
| Number of Trinity 'genes' / Corset 'clusters' | 442,924 | 140,592 |
| Number of transcripts | 552,851 | 227,791 |
| Mean transcript length (bp) | 587.6 | 1023.5 |
| N50 (bp) | 887 | 1668 |
| Total assembled bases (bp) | 324,828,327 | 233,136,858 |
|  |  |  |
| **BUSCOs** |  |  |
| Complete | 1051 (98.6%) | 1050 (98.5%) |
| Complete and single-copy | 360 (33.8%) | 362 (34.0%) |
| Complete and duplicated | 691 (64.8%) | 688 (64.5%) |
| Fragmented | 11 (1.0%) | 8 (0.8%) |
| Missing | 4 (0.4%) | 8 (0.7%) |
|  |  |  |
| **RNA-seq reads representation** |  |  |
| total number of paired reads | 147,396,767 | 147,396,767 |
| aligned concordantly ≥1 times | 124,862,703 (84.7%) | 123,763,176 (84.0%) |
| overall alignment | 143,652,889 (97.5%) | 142,385,277 (96.6%) |

**Table S3.** Annotation statistics for *Zelandoperla fenestrata* notum Trinity transcriptome

| **Annotation** | **Value** |
| --- | --- |
| **BLASTx (UniProt/Swiss-Prot)** | 153,455 (27.5% of transcripts) |
| **RNAMMER** | 72 (0.01% of transcripts) |
| **TransDecoder ORF finder** | 108,209 (19.4% of transcripts) |
| **BLASTp (UniProt/Swiss-Prot)** | 76,329 (70.5% of ORFs) |
| **Pfam** | 66,106 (61.1% of ORFs) |
| **TmHMM** | 13,822 (12.8% of ORFs) |
| **SignalP** | 6,944 (6.4% of ORFs) |
| **eggNOG** | 121,909 |
| **KEGG** | 117,107 |
| **BLAST gene ontologies** | 140,420 |
| **Pfam gene ontologies** | 41,821 |
